# Mechanical properties of the cement of the stalked barnacle *Dosima fascicularis* (Cirripedia, Crustacea)

**DOI:** 10.1098/rsfs.2014.0049

**Published:** 2015-02-06

**Authors:** Vanessa Zheden, Waltraud Klepal, Stanislav N. Gorb, Alexander Kovalev

**Affiliations:** 1 Faculty of Life Sciences, Core Facility Cell Imaging and Ultrastructure Research, University of Vienna, Vienna, Austria; 2 Zoological Institute: Functional Morphology and Biomechanics, Kiel University, Kiel, Germany

**Keywords:** adhesive, elastic modulus, hardness, tensile stress

## Abstract

The stalked barnacle *Dosima fascicularis* secretes foam-like cement, the amount of which usually exceeds that produced by other barnacles. When *Dosima* settles on small objects, this adhesive is additionally used as a float which gives buoyancy to the animal. The dual use of the cement by *D. fascicularis* requires mechanical properties different from those of other barnacle species. In the float, two regions with different morphological structure and mechanical properties can be distinguished. The outer compact zone with small gas-filled bubbles (cells) is harder than the interior one and forms a protective rind presumably against mechanical damage. The inner region with large, gas-filled cells is soft. This study demonstrates that *D. fascicularis* cement is soft and visco-elastic. We show that the values of the elastic modulus, hardness and tensile stress are considerably lower than in the rigid cement of other barnacles.

## 1. Introduction

Sessile marine organisms secrete adhesives which are cured underwater and which remain durable in the water [1,2]. The best-studied animals, producing a strong adhesive, are the invertebrates, such as barnacles, mussels and tubeworms [3].

Barnacles are among the most troublesome and dominant fouling organisms [4]. They settle as cypris larvae on any hard substratum, whether it is man-made such as ships and bridges or organisms such as crabs and turtles. After metamorphosis from the cyprid to the juvenile, both acorn and stalked barnacles usually produce only a thin layer of permanent adhesive, the so-called cement, by which they adhere to the substratum. The stalked barnacle *Dosima fascicularis* (Ellis and Solander, 1786) is exceptional, secreting a large amount of foam-like proteinaceous cement which is produced by the cement glands and passed through the stalk in a complex canal system. At the base of the stalk, the cement is extruded through pores in the cuticle. The cells enclosed in the cement contain gas [5–7], the nature of which remains unknown. It is assumed that it may be CO_2_, a by-product of metabolism, which is transported by the haemolymph and diffuses through the lining cells of the cement canals into the lumen of the ducts. The excretion of CO_2_ together with the cement is doubly advantageous for the animal: it causes the formation of gas-filled cells in the cement and contributes to the pH regulation in the haemolymph [8].

*Dosima fascicularis* mainly attaches to floating objects such as feathers, driftwood, seaweed and tar pellets [5,7,9,10]. As the animal grows, the amount of cement increases and can subsequently enclose any small substratum to which it adheres. With this so-formed float, *D. fascicularis* drifts passively in the neuston [11]. Other animals, such as marine snails of the family Janthinidae, also raft in the water but their float is of different origin and consists of bubbles of mucus [12,13]. It is assumed that the float is derived from an egg mass which is modified for buoyancy [12]. The precise composition of this material has not yet been analysed. Apart from being used for locomotion foam-like materials are also used for attachment and protection. Marine mussels such as *Mytilus californianus* and *Mytilus edulis* adhere to the substratum by means of the proteinaceous byssus. The adhesive plaque of the byssus resembles a solid foam with a spongy inner structure and a fibrous surface matrix [14–16]. According to Grayson [17, p. 531], a solid foam is defined as material ‘the apparent density of which is decreased substantially by the presence of numerous cells disposed throughout its mass'. The marine sandcastle worm *Phragmatopoma californica* builds protective tubes of solid foam [18] by gluing sand grains and parts of shells together with proteinaceous cement [19,20].

In addition, biofoams are frequently used by animals for the protection of brood. Examples are the nymphs of spittlebugs which secrete a froth consisting of a proteoglycan and glycoprotein complex. The froth surrounds their body and thus protects them, for example, from desiccation [21]. Fish, such as the armoured catfish, protect their eggs in floating foam nests consisting of mucus [22,23]. Tropical frogs produce proteinaceous foams for the protection of eggs and embryos against environmental challenges [24].

Little to nothing is known about the mechanical properties of the biofoams (e.g. [25,26]) and the mechanical qualities of the cement of stalked barnacles are still awaiting investigations. After the detailed documentation of the morphology of the cement apparatus and the cement [8] and the study of the biochemical composition of the cement of *D. fascicularis* [27], it is the aim of this study to investigate the mechanical properties of this adhesive. Studies investigating acorn barnacle cement show that its structure differs between different species and also within the same species depending on the substratum to which the animal is attached [2,28]. The adhesive may be fibrous, globular or sponge-like [1,2,29,30]. Accordingly, the mechanical properties of the cement differ. Among the best investigated qualities are adhesive tenacity, elastic modulus and hardness. On a hard substratum, the adhesive tenacity or removal stress of the temporary adhesive of the cyprid (which is searching for a suitable place for attachment) and of the permanent cement of the juvenile is almost the same (around 0.2 MPa). Considerably higher tenacity values (around 0.9 MPa) were found in the permanent adhesive of a settled cyprid and in the cement of the adult [31,32]. By using elastomeric coatings, the removal stress can be lowered and barnacles can easily be detached [30,33,34]. The substratum also influences the elastic modulus and hardness of the cement. These values are higher on non-metallic than on metallic substrata. In previous experiments, it was shown that acorn barnacles secrete more cement on low-energy polymeric surfaces to adhere firmly than on high-energy surfaces such as metal [2]. Compared with the cement of acorn barnacles, the *Dosima* cement has a different structure [8] and, according to the definition by Grayson [17], it is a solid foam. Its main function is to give buoyancy to the animal [9], apart from providing a reliable bond to the substratum. Therefore, it is to be expected that its mechanical properties differ from those of other barnacles whose only function is strong adherence to the substratum. In contrast to the thin and firm cement of acorn barnacles, the cement float of *D. fascicularis* appears soft and elastic.

In this study, both the elastic modulus and hardness of the cement were measured using micro-indentation. Furthermore, the cement float was pulled apart in a tensile test until it ruptured, and the tensile stress was compared with the stress measured in acorn barnacles at the pull-off from the substratum. The mechanical properties of the cement of *D. fascicularis* are interesting in comparison with those of acorn barnacles and in the context of the possible use of this adhesive in medicine and technology.

## 2. Material and methods

Individuals of *Dosima fascicularis* which had been washed ashore were collected on the northwest coast of Denmark. The cement was removed from the animals and stored in seawater with a 2% antibiotic antimycotic solution (Sigma-Aldrich, Vienna, Austria) prior to the analyses.

### 2.1. Electron microscopy

The cement was fixed in 2.5% glutaraldehyde in 0.1 mol l^−1^ sodium cacodylate buffer with 10% (w/v) sucrose at pH 7.3 for 2 h. For scanning electron microscopy, cross sections of the pre-fixed cement floats were rinsed in distilled water, air-dried and coated with gold by an Agar B7340 sputter coater (Agar Scientific Ltd, Stansted, UK). The samples were examined in a Philips XL 30 scanning electron microscope (FEI/Philips, Eindhoven, The Netherlands) at 15 kV.

For transmission electron microscopy small pieces of pre-fixed cement were post-fixed in 1% osmium tetroxide in 0.1 mol l^−1^ sodium cacodylate buffer for 2 h. The samples were dehydrated in a graded ethanol series, and acetonitrile was used as intermediate medium before embedding in Agar low-viscosity resin. Sixty-nanometre sections were cut on a Reichert Ultracut-S microtome (Leica Microsystems, Vienna, Austria), stained with 0.5% uranyl acetate and 2% lead citrate. The sections were viewed in a Zeiss EM 902 transmission electron microscope (Carl Zeiss Microscopy GmbH, Oberkochen, Germany) at 80 kV.

### 2.2. Micro-indentation experiments

**Figure 1.**
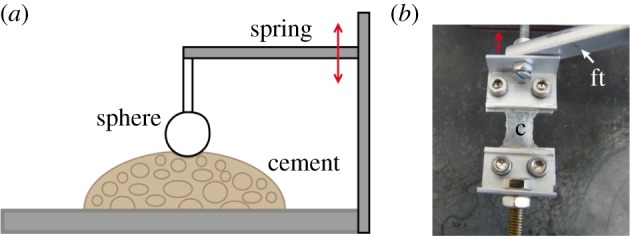
(*a*) Schematic drawing of the indentation experiment set-up. The glass sphere was attached to a spring, which was driven by a motor and indented the cement. (*b*) For the tensile experiment, a part of the outer region of a cement float (c) was fixed between two clamps. The upper clamp attached to the force transducer (ft) was pulling the sample with a constant speed, while the lower clamp was fixed.

The elastic (Young's) modulus and the hardness of the *D. fascicularis* cement were measured by micro-indentation. The cement floats were cut into halves. The almost smooth outer surface and the inner foam-like region were measured in seawater and distilled water at room temperature. Also the surface of the dried cement was measured. On each of these samples, which were fixed in a metal frame, three indentations were performed on different spots. For the experiments, the Basalt 01 microtribometer (Tetra GmbH, Germany) [35–37] was used. Indentations were performed using a glass sphere (3 mm diameter) fixed to a metal spring (figure 1*a*). The spring constant was calculated experimentally from the slope of the force–distance curve obtained on an aluminium block. The effective Young's modulus *E* and the hardness *H* of the cement samples were defined from the fit of the unloading part of the force–indentation depth curves according to the Hertz equation [38]
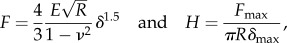
where *R* is the radius of the glass sphere, *ν* is the Poisson ratio, *δ* is the indentation depth caused by the applied force *F*, and *F*_max_ and *δ*_max_ are the maximal values of the applied force and indentation depth, respectively. The software Matlab v. 7.12.0 (The MathWorks, Inc., Natick, MA, USA) was used for the fit. Graphs, box plots and statistical tests were created in Sigma Plot v. 11.0 (Systat Software Inc., Bangalore, India, and San Jose, CA, USA). Statistical analysis was done with the non-parametric Kruskal–Wallis one-way analysis of variance on ranks with Dunn's multiple comparison procedure (significance set at *p* < 0.05). Data were fitted by linear regression and tested for normal distribution using the Shapiro–Wilk test.

### 2.3. Tensile test

In this experiment, whole cement floats as well as parts of the outer region of the cement float were used. These parts were cut into cubes of about 10 × 10 × 0.2 mm. The samples were then fixed by two clamps (figure 1*b*); one clamp was fixed on a force transducer FORT 100 (World Precision Instruments, Sarasota, FL, USA) which pulled the sample with a constant speed of 200 µm s^−1^. The cement was pulled in 5 mm steps, each followed by a resting period of around 8 s until the sample ruptured. Pulling force and distance were measured. Additionally, the strain at rupture was defined. Knowing the area (width multiplied by height) and the length of the cement samples, a stress–strain curve could be generated. The value of the stress *σ* was obtained from the applied force *F* divided by the cement cross-section area *A* (*σ* = *F*/*A*) and the strain *ɛ* from the extension *δ* divided by the length *l* of the cement sample (*ɛ* = *δ*/l). The graphs were created in Sigma Plot v. 11.0. The data for the relaxation were fitted by a single exponential function and tested for normal distribution using the Shapiro–Wilk test.

## 3. Results

### 3.1. Cement morphology

**Figure 2.**
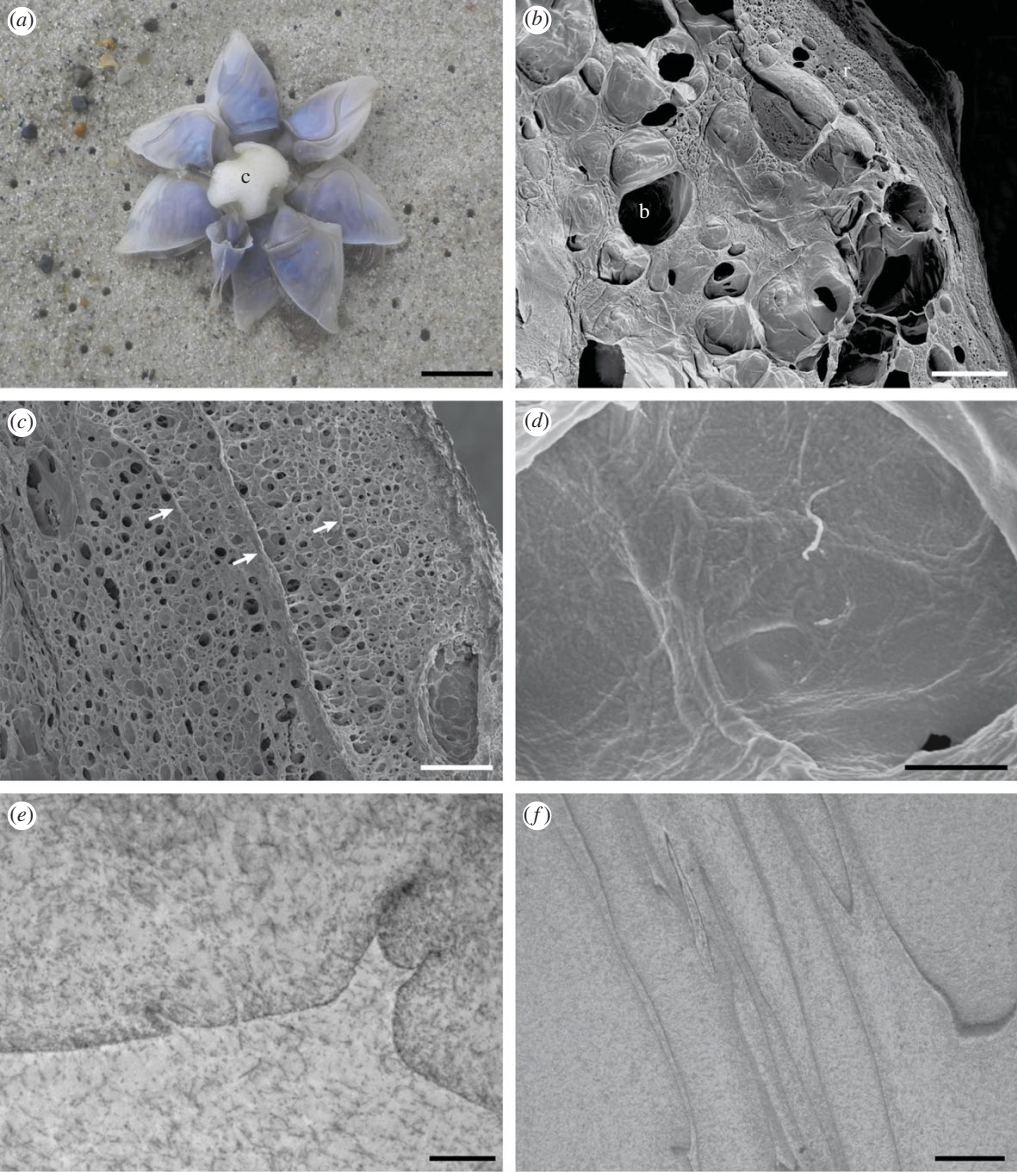
(*a*) Stranded aggregation of *D. fascicularis* connected by a single cement float (c). (*b*–*d*) Scanning electron micrographs. (*b*) Cross section of a cement float. The outer zone forming a rind (r) contained mainly small cells, whereas in the inner region of the float large cells (b) were dominant. (*c*) Higher magnification of the rind with narrow layers (arrows). (*d*) On the layers and the inner lining of the cells, the cement showed some roughness. (*e*,*f*) Transmission electron micrographs. (*e*) The cement had a fibrous structure. (*f*) In the region where the fibres were condensed, irregular lines were seen. Scale bars: (*a*) 1 cm; (*b*) 500 µm; (*c*) 100 µm; (*d*) 5 µm; (*e*) 2 µm; (*f*) 5 µm.

*Dosima fascicularis* occurred individually or in groups (figure 2*a*), producing a large amount of cement. The foam-like cement enclosed gas-filled cells of different size (figure 2*b*), which gave buoyancy to the animal. The cement was secreted in concentric layers around the stalk and the substratum to which the animal had attached. The outer layers, forming a kind of rind, were narrow and contained small cells (figure 2*c*). Inside the float were mainly large cells. Under the scanning electron microscope, the cement had a rough structure (figure 2*d*); under the transmission electron microscope it appeared fibrous with condensed zones, where the fibres aggregated (figure 2*e*,*f*). Some of these zones formed the frames of the cells.

### 3.2. Micro-indentation test

**Figure 3.**
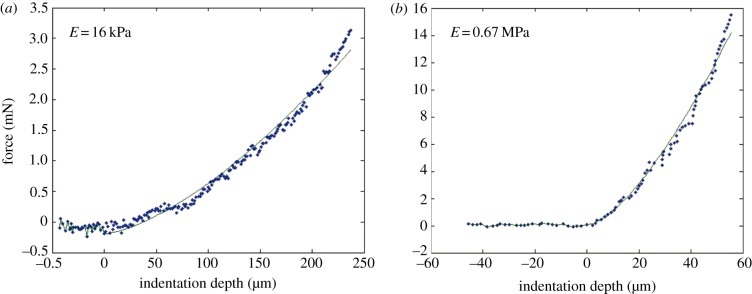
Typical force versus indentation depth curves of the cement of *D. fascicularis*. (*a*) The cement surface measured in seawater. (*b*) The surface of the dry cement. The solid line indicates the fit of the indentation data with the Hertz theory. *E*, elastic modulus. (Online version in colour.)

**Figure 4.**
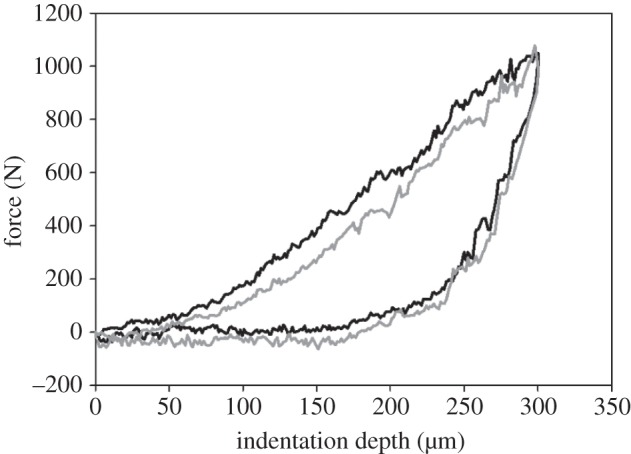
Two consecutive indentations, applied with the same force, measured on the same spot at the surface of a wet cement float of *D. fascicularis*. The second indentation (grey) demonstrated a visco-elastic-plastic deformation when compared with the first indentation (black).

Typical curves of loading force versus indentation depth of the wet and the dry cement are shown in figure 3*a*,*b*. The force–indentation depth curves of two consecutive indentations at the same spot on the float provided information about visco-elastic-plastic deformation of the cement during the loading/unloading process (figure 4). The plastic deformation was seen as a shift of the second indentation curve (grey) relative to the first indentation curve (black). The large difference in the loading and unloading parts of the indentation curves demonstrated the pronounced viscose properties of the float. However, the main mechanical response of the float on the indentation was elastic.

**Figure 5.**
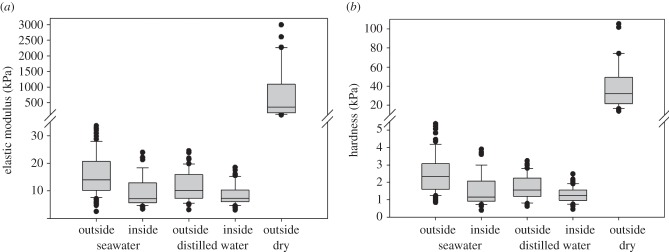
The elastic modulus (*a*) and the hardness (*b*) of the surface and inner region of the cement float of *D. fascicularis* measured in seawater and distilled water as well as of the surface of the dry cement. Box plots show the median value (line), the ends of the boxes define the 25th and 75th percentiles and the error bars the 10th and 90th percentiles. The outlines are illustrated as black dots. The difference between the wet and dry cement was obvious, therefore only the differences between the different regions under wet conditions were analysed using Kruskal–Wallis one-way analysis of variance on ranks. (*a*,*b*) *p* < 0.001.

The elastic modulus of the cement surface measured in seawater was 16.4 kPa ± 8.8, whereas in distilled water it was 11.6 kPa ± 5.3. In the inner region, the modulus was 9.3 kPa ± 5.3 measured in seawater and 8.5 kPa ± 3.6 in distilled water. Statistically, there was a significant difference between the different regions of the cement in the two media (*p* < 0.001, Kruskal–Wallis test). However, the pairwise comparison revealed that the inner regions were not significantly different. The dry cement had a much higher Young's modulus (0.76 MPa ± 0.87) than the cement under wet conditions (figure 5*a*).

The hardness of the cement surface measured in seawater was 2.5 kPa ± 1.2, whereas in distilled water it was 1.7 kPa ± 0.7. In seawater, the inner region had a hardness of 1.5 kPa ± 0.9; in distilled water, 1.3 kPa ± 0.4. As for the elastic modulus, the hardness differed significantly between the different regions and media (*p* < 0.001, Kruskal–Wallis test), but again the pairwise comparison showed that there was no significant difference between the inner regions of the float in seawater and distilled water. The dry cement had the highest hardness values (39.0 kPa ± 23.2) (figure 5*b*).

**Figure 6.**
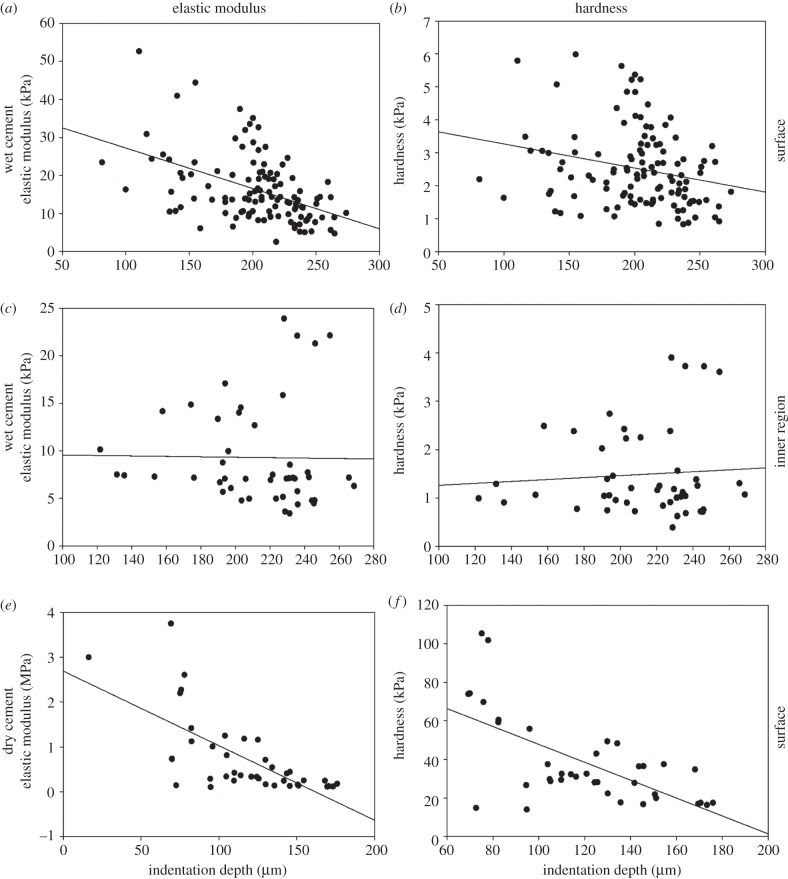
Correlation between the indentation depth and the elastic modulus (*a*,*c*,*e*). Correlation between the indentation depth and the hardness (*b*,*d*,*f*). (*a*,*b*) Surface of the wet cement (in seawater, *n* = 116). (*c*,*d*) Inner region of the wet cement (in seawater, *n* = 46). (*e*,*f*) Surface of the dry cement (*n* = 40). The data were fitted by linear regression and tested for normal distribution using a Shapiro–Wilk test. (*a*) *y* = 37.85–0.11*x*, *R*^2^ = 0.22, *p* < 0.0001; (*b*) *y* = 4.002–0.007*x*, *R*^2^ = 0.06, *p* < 0.0001; (*c*) *y* = 9.77–0.002*x*, *R*^2^ = 0.0002, *p* < 0.0001; (*d*) *y* = 1.063 + 0.002*x*, *R*^2^ = 0.006, *p* < 0.0001; (*e*) *y* = 2.69 − 02*x*, *R*^2^ = 0.47, *p* = 0.07; (*f*) *y* = 94.18 − 46*x*, *R*^2^ = 0.43, *p* = 0.1 (*R*^2^ = coefficient of determination).

The elastic modulus and the hardness were higher at the surface than in the inner region of the wet cement. With an increasing indentation depth, the elastic modulus (*R*^2^ = 0.22, Shapiro–Wilk test) and the hardness (*R*^2^ = 0.06, Shapiro–Wilk test) decreased (figure 6*a*,*b*). In the inner region of the cement float, no correlation between the indentation depth and the elastic modulus (*R*^2^ = 0.0002, Shapiro–Wilk test) or hardness (*R*^2^ = 0.006, Shapiro–Wilk test) was observed (figure 6*c*,*d*). In the dry cement, the values of the elastic modulus (*R*^2^ = 0.47, Shapiro–Wilk test) and hardness (*R*^2^ = 0.43, Shapiro–Wilk test) decreased significantly with an increasing indentation depth (figure 6*e*,*f*).

### 3.3. Tensile test

**Figure 7.**
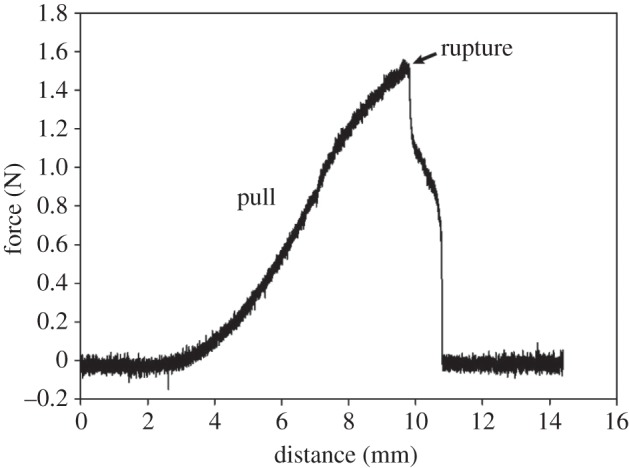
The curve shows a force–distance dependence during pulling of the cement (in this case, a parallelepiped-shaped sample of the outer region of the float was used) until it ruptured.

**Figure 8.**
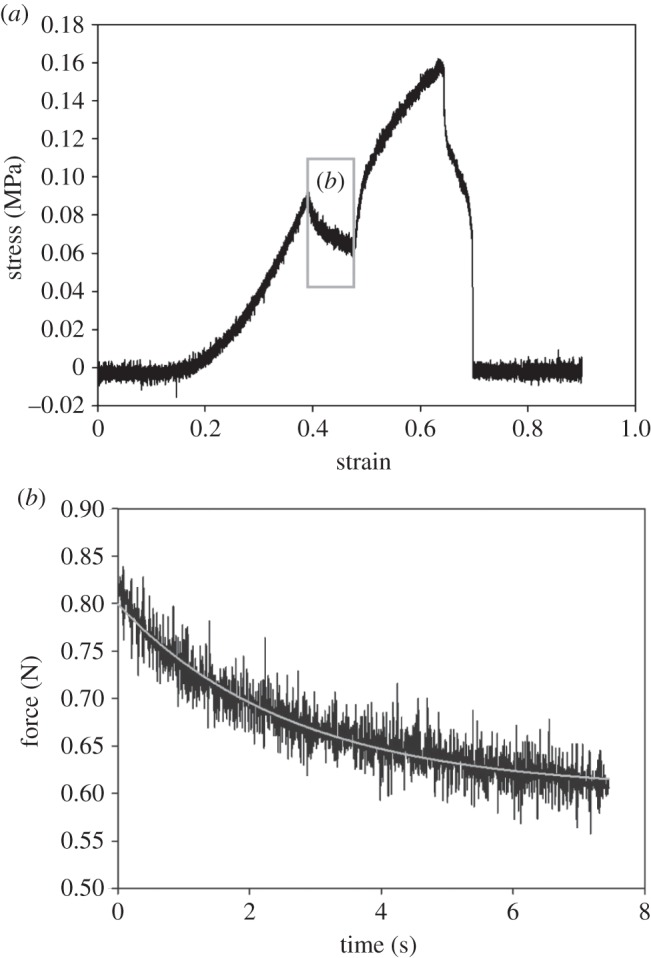
(*a*) The stress–strain curve obtained in the pulling experiment showed relaxation (see (*b*)) of the cement, when pulling was stopped for around 8 s. (*b*) Relaxation (decrease in the interacting force) of the cement, lasting for several seconds, was observed during the resting period. The relaxation process was strongly pronounced during deformation of the cement. It was represented by a single exponential function and tested for normal distribution using the Shapiro–Wilk test. *f* = 0.6–0.2 × 10^–0.4*x*^, *R*^2^ = 0.88, *p* < 0.0001 (*R*^2^ = coefficient of determination).

During pulling, the cement was elastically extensible to the point of rupture (figure 7). The tensile stress of the cement was below 0.2 MPa (figure 8*a*). During the resting period, the force decreased, indicating slow relaxation of the material (figure 8*a*,*b*). This proved the visco-elastic properties of the cement.

## 4. Discussion

Many organisms use adhesives for a variety of purposes, e.g. for attachment, defence or protection [18,39,40]. In marine animals, the attachment can be permanent as in mussels, transitory as in turbellarians, or temporary as in echinoderms [41]. Barnacles use both temporary and permanent adhesives during their life cycle. The last larval stage, the cyprid, uses temporary adhesion to explore the substratum before settlement and the adult is permanently attached [32]. According to Yule & Walker [31], the cyprid used a low-bond-strength cement in comparison with the higher-bond-strength cement of the adult.

Normally barnacles deposit a thin layer (a few micrometres thick) of firm adhesive on high-energy surfaces. Only when they adhere firmly on polymeric substrata with low energy do they produce a thicker cement layer with a sponge-like structure. Interestingly, this spongy cement is harder and has a higher elastic modulus than the firm cement [2]. In contrast to all other barnacles, *D. fascicularis* produces a large amount of foam-like cement which contains gas-filled cells. The cement is secreted in concentric layers around the stalk and the attached substratum [8]. It is known that the layered structure of barnacle cement is the result of cyclic secretion during the growth of the animal [30,42,43]. Sun *et al*. [30] described the multi-layered structure of the adhesive plaque of *Balanus eburneus* and *Balanus variagatus*. In these species, the elastic modulus, in the range of 0.01–100 MPa, increased from the outer to the inner layer. In contrast to the *Balanus* cement, the surface of the *Dosima* cement had a higher elastic modulus and was harder than the inner region (figure 6). A reason for this may be that the salts in the seawater hardened the surface of the cement. In experiments, we could show that the elastic modulus and hardness of the cement surface were higher in seawater than in distilled water. In addition, the outer narrow layers formed a rind, containing only small cells (about 11 µm in diameter) compared with the inner region where the elongate cells (up to 2460 µm in length) predominated [8]. This structure would give greater mechanical stability and stronger protection to the surface region. The rind, as the interface with the environment, would be prone to any mechanical- or UV damage and possible dehydration. Similar structural and mechanical characteristics were also observed in the cement of *P. californica*, where the smallest cells were at the interface with the surrounding water [18]. These authors suggested that the highest elastic modulus would be at the interface. Also the inner spongy plaque matrix of the mussel *M. edulis* became increasingly dense towards the outside [14]. This seems to be a common phenomenon in the adhesives of aquatic animals.

The cement of barnacles was generally fibrous [8,29,44–46] and visco-elastic [2,30]. Visco-elasticity is known to be a property of many, if not all, biological materials [47]. This term has been used to describe natural fibrous composites, such as the cuticle of the attachment pads of Orthoptera [35,37]. Also non-fibrous materials such as those of echinoderm tube foot discs [48] and the adhesive gels of gastropods [49] have visco-elastic properties. Visco-elasticity of the cement of *D. fascicularis* may be necessary to protect the gas-filled cells inside the float from rupture by any fast and strong mechanical impact, for example water current and waves.

Most values of the elastic modulus of the wet cement of *D. fascicularis* were in the range of 5–20 kPa. Investigations of the cement of the acorn barnacle *Amphibalanus* spp. showed that the elastic modulus of the cement was higher than that of *D. fascicularis*. The modulus of elasticity of the cement of *A. amphitrite* measured by Ramsay *et al.* [50] with punch test apparatus was between 2.9 and 6.5 GPa. Sullan *et al.* [1] performed atomic force microscopy nanoindentations on different structures of the cement of the same species and got lower values of the elastic modulus ranging from 0.2 to 90 MPa. The difference in these results may be due to the different methods used. Sangeetha & Kumar [2] analysed the cement of *A. reticulatus* growing on metallic and non-metallic substrata, using a nanomechanical testing system. The hardness and the elastic modulus of the cement were higher on non-metallic (*H* = 52.6 MPa and *E* = 1.2 GPa) than on metallic (*H* = 8.7 MPa and *E* = 0.4 GPa) substrata and again higher than those of the *Dosima* cement. These authors reported that barnacles needed more cement to adhere firmly to non-metallic substrata than to metal and that detachment of barnacles from metallic surfaces was generally more difficult than from non-metallic ones.

The acorn barnacles described above had a calcareous base. It cannot be ruled out that parts of the hard calcareous base were included in the measurements. In our experiments, only the pure cement, free of any animal tissue and free of any substratum, was investigated. In the indentation experiments, we selected indentation depths at least 10-fold lower than the sample thickness, in order to prevent contribution of the stiff support to the results of our measurements.

Our results revealed that the *D. fascicularis* cement is a soft biological material. Its elastic modulus was in the same range as that of other animal adhesive structures such as the tube foot discs of echinoderms (3–140 kPa) [48], the adhesive pads of ensiferan insects (25–100 kPa) [37] and the adhesive secreted by the Australian frog *Notaden bennetti* (170–1035 kPa) [51]. The adhesives secreted by the serpulid *Hydroides dianthus* (about 3 GPa [52]) and the byssal threads of *Mytilus* mussels are very hard marine biomaterials. The protective outer cuticle of the byssal threads of *M. californianus* and *M. galloprovincialis* had a modulus of about 1.7 GPa and a hardness of around 0.1 GPa. The elastic modulus and the hardness of the cuticle were four to six times greater than that of the inner collagen core of the byssal threads [25].

The dry cement of *D. fascicularis* had an elastic modulus of approximately 0.8 MPa and was thus much harder than the wet cement. It is known that dehydration hardened the originally soft barnacle adhesive [30]. Desiccation also hardened biological materials, such as insect cuticle or bones [53,54].

The adhesive strength or removal stress of acorn barnacles was, like the elastic modulus and the hardness, influenced by the substratum. Unlike the elastic modulus and hardness, the tenacity (adhesive strength) was higher on ‘natural’, hard surfaces (around 0.9 MPa) than on polymeric substrata (less than 0.1 MPa) [32,33,55]. *Dosima fascicularis* mostly settled on small, organic floating objects, which were gradually enclosed in the cement float. Therefore, the cement could not be removed from the substratum and the removal stress could not be measured. In order to compare the acorn barnacle cement with that of *D. fascicularis*, the *Dosima* cement was pulled apart until rupture. The tensile stress determined in our experiment was below 0.2 MPa. Accordingly, the tensile stress of the *Dosima* cement was lower than the removal stress of the cement of acorn barnacles attached to a natural stiff substratum, but it was slightly higher than that of barnacles attached to polymeric substrata.

## 5. Conclusion

The cement of *D. fascicularis* is a soft biological material and like that of other barnacles fibrous and visco-elastic. The values of elastic modulus, hardness and tensile stress are much lower than in the rigid cement of acorn barnacles investigated so far. A physical explanation for these differences is the foam-like structure of the *Dosima* cement caused by the gas-filled cells. An ecological explanation could be the differing living conditions of acorn barnacles and *Dosima* with the partly different use of the adhesive. In contrast to the gregariously settling acorn barnacles, which are firmly attached to the substratum, *D. fascicularis* occurs either singly or in small numbers attached to floating objects or drifting through the sea with a cement float. For this lifestyle, the *Dosima* cement has to be able to withstand mechanical impact in the water, it must not be hard as in other barnacles and—very importantly—the float must be impermeable to water and gas. The great elasticity enhanced by the foam-like structure gives the cement damping properties. In addition, this special structure of the cement is more economical for the animal than a solid structure. It saves material and thus energy.

The shock-absorbing properties combined with the expected biocompatibility of the *Dosima* cement make it interesting for possible applications in orthopaedics. Its structure and the assumed biodegradability make it perfectly suitable as three-dimensional scaffolds for tissue growth and wound healing like other biofoams [56,57]. Besides the possible application in medicine, the *Dosima* cement could also be used in technology. Its foam-like weight-saving structure in conjunction with the fact that the cement cures and is durable underwater could make it an appropriate material for construction works in a wet environment.
